# Childhood maltreatment is associated with distrust and negatively biased emotion processing

**DOI:** 10.1186/s40479-020-00143-5

**Published:** 2021-02-03

**Authors:** Johanna Hepp, Sara E. Schmitz, Jana Urbild, Kathrin Zauner, Inga Niedtfeld

**Affiliations:** 1 Department of Psychosomatic Medicine and Psychotherapy, Central Institute of Mental Health, Medical Faculty Mannheim/Heidelberg University, J5, 68159 Mannheim, Germany; 2 University of Mannheim, Mannheim, Germany

**Keywords:** Childhood maltreatment, Adverse childhood experiences, Childhood trauma, Distrust, Trust game, Emotion processing, Post-traumatic stress disorder

## Abstract

**Background:**

Cognitive models of post-traumatic stress disorder (PTSD) propose that trauma entails cognitive alterations of increased distrust and perceived threat from others. We tested whether these predictions also hold in individuals with varying levels of childhood maltreatment (CM), which is much more prevalent than traumatic events as required for a PTSD diagnosis. We hypothesized that higher levels of CM would entail greater distrust and perceived threat, and that distrust would be more change-resistant in participants with more CM.

**Methods:**

The study was pre-registered; the pre-registration protocol, data, and code are available at https://osf.io/pufy2/. We recruited 549 participants (*M* age = 29.2, 74.5% women) for an online study via websites related to CM, Borderline Personality Disorder, and via snowball method on social media. Participants self-reported their level of CM on the childhood trauma questionnaire (CTQ). Next, they played two rounds of a hypothetical distrust game, indicating the perceived trustworthiness of avatars by way of estimating expected monetary deductions from them (i.e. higher amounts indicating greater distrust). After the first round, we provided participants with the feedback that very little money was taken from them. We expected those with more CM to be less responsive to the positive feedback and to adapt their estimates less in the subsequent round. Following the distrust game, participants completed an emotion rating task in which they rated the emotional expressions of 60 faces on a scale from ‘very negative’ to ‘very positive’. We included angry, fearful, and happy facial expressions, and expected individuals with higher CM levels to provide more negative ratings. We conducted linear mixed effects models with random intercepts for raters and stimuli (crossed), and modelled random slopes for all within-person predictors.

**Results:**

As hypothesized, higher levels of CM were associated with higher levels of distrust and a weaker decrease in distrust following positive feedback. Further supporting our hypotheses, individuals with higher levels of CM showed more negatively shifted emotion ratings.

**Conclusions:**

Increased distrust and perceived interpersonal threat following trauma, as proposed in cognitive models of PTSD, likely also apply to individuals with CM, following a dose-response relationship. We discuss clinical implications of considering any level of CM as a potentially relevant treatment-factor, even when a trauma-related disorder is not the main diagnosis, and propose future research avenues.

**Supplementary Information:**

The online version contains supplementary material available at 10.1186/s40479-020-00143-5.

## Introduction

Child maltreatment (CM) is a prevalent and debilitating phenomenon that serves as a risk factor for a multitude of negative outcomes, spanning both somatic and mental health problems [1]. It is defined as “any act or series of acts of commission or omission by a parent or other caregiver that results in harm, potential for harm, or threat of harm to a child” (p. 11 [2]). This includes, for instance, emotional neglect (e.g., parents that are not available to comfort their child when in distress), emotional abuse (e.g., parents that insult or devalue their child), physical neglect (e.g., parents that fail to provide food, clothing, or medical care for their child), physical abuse (e.g., parents that beat their child), and sexual abuse [3]. The prevalence for CM varies considerably depending on the country of assessment and the specific types of maltreatment that are considered, but estimates from most countries indicate that 40 to 50% of the population experienced some form of CM (see [4] for a review). The prevalence of psychopathology is elevated among individuals with CM (e.g. [5]) which is reflective of a global and strong association between CM and general psychopathology [1]. In addition to mental health consequences, CM is also predictive of poorer psychosocial adjustment and socioeconomic status later in life (for a review see [6]). Beyond the strong association between CM and psychopathology in general, CM is a specific risk factor for the development of post-traumatic stress disorder (PTSD, e.g. [7]).

Cognitive models of PTSD [8, 9] propose a range of cognitive alterations that can follow traumatic events. In detail, trauma is theorized to lead to negative cognitions regarding trust (e.g., ‘I can trust no one’), safety/threat (e.g., ‘Most people and contexts are dangerous’), power (e.g., ‘I have no control over what happens to me’), self-regard (e.g., ‘I am forever changed’), and intimacy (e.g., ‘I cannot be close to anyone’) [9]. While these cognitive alterations have been demonstrated for severe types of trauma that entail a threat to one’s life or well-being, which is required for a formal diagnosis of PTSD following DSM-5 [10], it is currently unclear whether similar cognitive alterations also occur following CM, which is not necessarily life-threatening (e.g., in the case of emotional neglect). However, negative cognitions regarding trust and safety may also underlie the restricted psychosocial functioning that is associated with CM [11]. In turn, impairments in social functioning and a lack of social support are a known risk factors for the development of mental disorders [12], including PTSD [13].

Evidence on increased distrust and perceived threat following CM could substantially inform treatment approaches, especially when patients are in treatment for conditions other than a trauma-related diagnosis, but with CM as an additional factor to consider. Therefore, we aimed to test the association between CM and two areas of negative cognition described in cognitive models of PTSD [8, 9]. We selected the areas of *distrust* and subjective *threat*, as we deemed them highly relevant for overall functioning, arguing that a lack of trust in others and perceiving others as threatening would entail a lack of psychosocial integration and functioning that spans private and professional relationships (see [1]).

The previous evidence on trust and perceived threat in the context of CM is somewhat mixed and based on a wide range of different samples and varying study designs. A number of cross-sectional studies have demonstrated that individuals who experienced CM tend to self-report lower levels of trust in others, and this was shown across various age groups. One study assessed children aged 8 to 12, and found that children who had suffered sexual abuse reported significantly lower interpersonal trust than non-exposed children did [14]. Adding to this, physical abuse was positively associated with the number of distrust-related cognitions adolescent participants noted in their study diaries [15]. In samples of young adults, CM was negatively associated with self-reported general trust [16, 17]. Moreover, there is a small number of experimental studies that assessed how individuals who experienced CM perform in the *trust game*. The trust game is an economic exchange game in which participants play with an interaction partner, make, and receive monetary contributions. Lower trust is indicated by smaller monetary contributions or investments in the interaction partner (i.e., expecting that one’s contribution will not be repaid and that one’s trust will be broken). One study found that girls with and without a history of physical or sexual abuse showed similar monetary contributions in the trust game [18]. Contrasting this, adolescent girls with CM showed a lower ability to learn which individuals are trustworthy than non-exposed girls in the trust game. That is, they updated their ratings less in response to changes in contributions made by the interaction partner [19]. Importantly, this effect was stronger for individuals who reported more maltreatment events, suggesting that the learning deficit increased, the more CM one reported.

In light of the small number of experimental studies that directly address CM in relation to trust, it appears helpful to also consider evidence from studies using PTSD samples. It is likely that a substantial proportion of individuals in the PTSD groups of these studies would have experienced CM, whereas others might have experienced trauma later in life or trauma of a non-interpersonal nature. The PTSD evidence shows that individuals with PTSD indicated lower trust than HCs when playing with a cooperative partner, but similar levels of trust when playing with an uncooperative partner [20]. With regard to learning in the trust game, individuals with PTSD reduced their investments more strongly than HCs when an interaction partner switched from cooperative to uncooperative behavior and maintained this lower investment rate for longer [21]. At the same time, when considering all trials, PTSD participants showed lower *overall* learning rates than HCs. That is, PTSD participants did not adapt their investments as contingently upon the interaction partner’s behavior as the HC group did.

Regarding the association between CM and increased perception of threat, there is evidence for an altered processing of *faces* that signal threat. This includes both faces showing an angry expression (acting as a threat cue in the sense that the angry person that is displayed poses a potential threat) and a fearful expression (signaling that the displayed person may have detected a threat in the environment). A first group of studies assessed samples of children or adolescents. Five studies found that children who experienced maltreatment in the past showed an atypical processing of angry faces. In particular, exposed children showed a response bias for angry faces, such that they tended to match emotional situations of any valence to a picture of an angry face [22] and used anger labels significantly more often than non-exposed children to describe displayed emotions [23]. Moreover, exposed children were more likely to interpret ambiguous facial expressions as angry [24], to correctly identify facial expressions of anger based on less perceptual information [25], and to attend to angry faces more than non-exposed children did [26]. In addition, children who experienced maltreatment displayed significantly faster reaction times than controls when labeling emotional facial expressions and this result was most pronounced for fearful faces [27].

Beyond this evidence in children, adolescents with a history of CM showed increased sensitivity in the detection of angry expressions at lower levels of emotional intensity and preferential attention to angry faces in a dot-probe task [28]. Studies with adults found that individuals who experienced maltreatment during childhood showed a lower recognition accuracy for positive and neutral emotions [29], and a higher accuracy for negative emotions [30]. Moreover, two studies found that CM was associated with interpreting neutral expressions as angry, contemptuous, or fearful [31, 32]. Contrary to these results, another study found no alteration of fear processing [33], or even a lower accuracy for the recognition of fear [34] in individuals who experienced CM. These discrepancies might be due to variations in sample characteristics such as age, gender, psychopathological status, and trauma type, as well as experimental task variability.

In sum, the empirical evidence suggests that decreased trust and increased threat perception, as postulated in cognitive models of PTSD [8, 9], may also affect those with a history of CM, many of which do not meet criteria for a PTSD diagnosis. However, we argue that previous studies do not provide a conclusive test of this association, as they are limited by a number of factors. First, most previous studies focused on a specific subtype of CM and did not assess the whole range of maltreatment *types*. Second, almost all previous studies have relied on a case-control design, comparing individuals who met a certain threshold-level of CM with individuals without a history of maltreatment. Thus, previous studies have mostly neglected to assess effects of the *intensity* of CM, and thus considered individuals with high levels of maltreatment that may largely overlap with the PTSD population. This way, some *types* of maltreatment (e.g. emotional neglect) and milder *intensities* of maltreatment remain largely unexplored. At the same time, epidemiological studies suggest that milder intensity of CM may be particularly prevalent and that CM is distributed somewhat continuously in the general population, warranting a more continuous assessment in studies also [1, 5, 35]. Third, many of the previous studies were conducted in samples of children or adolescents. This renders unclear whether CM is associated with increased distrust and threat perception later in life. In other words, in samples of children, the time-point when maltreatment occurred is much more proximal and therefore possibly more impactful than when considering adults for whom experiences of CM lie several decades in the past. Considering a broader age range would provide more information regarding the stability of CM effects on cognitions. Following from these limitations, we aimed to test whether predictions regarding increased distrust and threat perception (cognitive model of PTSD, [8, 9]) hold in adults with varying levels and types of CM.

### The present study

The aims, hypotheses, design, and analyses for this study were pre-registered prior to data collection and the pre-registration protocol is available at https://osf.io/gcep6, as is all the data and analysis code for the study. Data for this study was collected online due to feasibility reasons^1^ and to obtain a large sample of participants with varying levels and types of CM, including both individuals recruited from the general public and through channels that target a more clinical audience (e.g., our clinic website). The first aim of the study was to test whether CM is associated with increased distrust in others. This is postulated as a social-cognitive consequence of trauma [8, 9], and has been shown in several empirical studies [14–17, 19], though so far only for restricted age groups and trauma types and often only by self-report. We assessed distrust with a multi-round, hypothetical *distrust game* (adapted from [36]) and incorporated a positive feedback condition. We derived the following hypotheses for the distrust game. *H1a:* CM predicts increased levels of distrust in the distrust game. *H1b:* CM predicts a diminished adaptation to positive feedback in the distrust game. In other words, we expected individuals with higher levels of CM to be less responsive to positive feedback. This was based on theoretical assumptions that cognitive schemas of distrust should be relatively stable across situations and not easily modified (e.g. [37]), and preliminary empirical evidence that points to decreased learning abilities for individuals with CM in the trust game [19]. Here, we were specifically interested in the effects of *positive* feedback, because correcting negative cognitive schemas through new, positive experiences is a mechanism that many therapeutic approaches encourage (e.g., making corrective, positive experiences in the present with interaction partners that are trustworthy and safe).

The second aim of the present study was to assess the association between CM and the perception of threat. Like distrust, social-cognitive alterations regarding increased perception of threat are postulated to follow trauma [8, 9]. However, this has yet to be tested in an adult sample with varying levels and types of CM, as past research heavily relied on samples of children [22–27]. To test this, we employed facial expressions that signal threat, including anger (signaling possible threat from the displayed individual) and fear (signaling possible threat in the environment). We presented participants with a range of faces displaying angry and fearful expressions of varying intensity, as well as happy faces for comparison, and asked them to rate the displayed emotions ranging from “very negative” to “very positive”. Based on cognitive models of PTSD [8, 9] and previous empirical evidence [30–32], we hypothesized that participants with more CM would rate facial expressions more negatively *(H2)*.

## Method

### Procedure

Ethics approval for this study was granted by the Medical Ethics Committee II of the medical faculty Mannheim at Heidelberg University (protocol no. 2018-588 N-MAb). Participants completed the study online. The study was constructed using the free online survey and experiment builder Soscisurvey (www.soscisurvey.de [38]). After following the study link, participants first received detailed information about the study content and duration, including information on data protection, and a disclaimer that the study included a potentially stress-inducing questionnaire about childhood trauma. Participants were ensured that all their data would only be stored anonymously and that no connection to their person could be drawn. They had the opportunity to download the study information as a separate pdf file and were encouraged to save this document and take sufficient time to read it in detail. Contact addresses of the first and last author were provided in case participants had any further questions or concerns prior to or following participation. After the first screen with the study information, participants were forwarded to a page where they indicated consent by clicking a checkbox. On this page, participants were also reminded that they were not allowed to participate in the study if they had previously participated, and that they had to be at least 18 years old. Participants who did not indicate consent and being at least 18 years of age by selecting the checkbox were *not* forwarded to the study slides.

After giving consent, participants provided demographic details and filled out the CM questionnaire. Next, they completed a two-round distrust game, followed by an emotion rating task. At the end of the study, participants were given the opportunity to provide their email address if they would like to a) participate in a raffle, b) receive information about the study results, or c) leave their address to receive notification about opportunities to participate in future studies. We saved and stored Email addresses separately from the study data (via a built-in feature in soscisurvey) to ensure participants’ anonymity. Participants were not compensated, but offered the opportunity to participate in a raffle for one of twenty 10 Euro Amazon gift cards.

### Participants

We advertised the study as a study assessing the impact of “different childhood experiences” on “emotion processing” and left participation open to everyone above the age of 18, since we were aiming to assess the whole range of CM in a dimensional fashion. To ensure that the sample would also comprise participants with higher levels of CM, we spread the study link on social media via snowball method and posted in online forums and facebook groups related to topics of CM and Borderline Personality Disorder (because individuals with BPD exhibit high rates of CM [39]). Additionally, we recruited participants through the facebook pages and websites of two clinical research groups within our institution (Central Institute of Mental Health, Mannheim, Germany) that study BPD and CM.

We recruited a total of 574 participants. Based on pre-registered exclusion criteria, we excluded three participants because they spent less than 2 s on answering each item of the CM questionnaire. We further excluded 14 participants who showed zero variance on any of the dependent variables, one person who indicated they had less than “good” German language proficiency, and seven participants who had missing values on the CM questionnaire. These individuals had to be removed from the analyses because analyses relied on the sum score of the CM inventory.

After applying exclusion criteria, the final sample comprised 549 participants. The majority of participants (*n* = 389) was recruited via snowball method on social media such as facebook, Whatsapp or Instagram. Seventy-seven participants were recruited through facebook groups and online forums dedicated to topics surrounding CM and BPD, and 46 participants were recruited through the facebook pages and websites of two clinical research groups at the CIMH, Mannheim, Germany. The remaining participants were recruited by Instagram influencers with channels on mental health topics, who shared our study link. Participants in the final sample were 18 to 70 years old, with the mean age reflecting that the sample comprised mostly young adults (*M* = 29.2, *SD* = 12.2). Sample diversity was restricted for gender, nationality and migration background, as the majority of the participants were women (74.5%), who lived in Germany (92.0%), and had no migration background (80.1%). Furthermore, the majority of participants held a university level entrance degree or university degree (78.7%). The sample was diverse with regard to monthly income and employment, with 51.0% of participants being students enrolled in school, university or in an apprenticeship, 32.1% currently employed, and the remaining participants indicating they were on disability, retired, or other (16.9%). We present detailed demographic data for all categories in Table 1.

**Table 1:** Sociodemographic Data

	*N = 549*	
	*n*	%
*Gender*		
Female	408	74.32
Male	127	23.10
Diverse	4	0.73
Did not want to assign their gender	10	1.82
*Highest educational level*		
No degree	4	0.73
Still in school	16	2.91
High school degree ^a^	44	8.01
University level entrance degree ^b^	319	58.11
Completed apprenticeship	48	8.74
Bachelor’s degree	45	8.20
Graduate degree	73	13.30
*Employment status*		
Unemployed	18	3.28
Currently on sick leave or disability	12	2.19
High school student	21	3.83
In apprenticeship	54	9.84
Undergraduate student	179	32.60
Graduate student	33	6.01
Employed	170	30.79
Self-employed	20	3.64
Retired	25	4.55
Other	17	3.10
*Monthly net income*		
< 500€	135	24.59
500–1000€	153	27.87
1000–1500€	71	12.93
1500–2000€	50	9.11
2000–2500€	45	8.20
2500–3000€	22	4.01
> 3000€	30	5.46
Did not want to indicate their income	43	7.83
*Home country*		
Germany	505	91.99
Austria	29	5.28
Switzerland	9	1.64
Other	6	1.09
*Migration background*		
No migration background	440	80.15
First-generation migrant	27	4.92
Second-generation migrant	74	13.48
Third-generation migrant	37	6.74
*Level of German language proficiency*		
Native	512	93.26
Very good	30	5.46
Good	7	1.28

^a^ Includes the German “Hauptschul−/Volksschulabschluss” and “Realschulabschluss”

^b^ German “Abitur”

With regard to clinical characteristics of the sample, 14.9% of participants stated they were currently in psychotherapy and 32.4% indicated that they had been in treatment at least once in the past. Furthermore, 13.3% indicated they were currently taking psychotropic medication, and 36.8% of participants reported that, at one point, they had received one or multiple diagnoses from a physician or mental health provider. The most commonly endorsed diagnoses were depression (20.6%), PTSD (14.0%), and BPD (12.6%). Details on these self-reported clinical characteristics are presented in Table 2.

**Table 2:** Self-reported mental health data

	*N = 549*	
	*n*	%
*Current mental health diagnosis*		
Yes	202	36.80
No	336	61.20
Not indicated	11	2.00
*Psychotherapy*		
Currently in psychotherapy	82	14.94
One past psychotherapy	99	18.03
More than one past psychotherapy	79	14.39
No previous psychotherapy	279	50.82
Not indicated	10	1.83
*Psychotropic medication*		
Currently on psychotropic medication	73	13.30
Past psychotropic medication	36	6.56
No previous psychotropic medication	439	80.00
Not indicated	1	0.18

### Material

#### CM questionnaire

Type and severity of adverse childhood experiences were assessed with the Childhood Trauma Questionnaire (CTQ [40]). The CTQ is a self-report measure that retrospectively assesses CM before age 18. It comprises 25 items with five subscales for different types of CM (emotional neglect, emotional abuse, physical neglect, physical abuse, and sexual abuse). Each item is rated on a five-point Likert-type scale, ranging from 1 = ‘not at all’ to 5 = ‘very often’, and all individual items are summed up to create a total score. The total score ranges from 25 to 125 and the five subscale scores range from 5 to 25, with higher scores indicating more severe childhood trauma. The CTQ has demonstrated good psychometric properties in clinical and nonclinical samples, including high retest reliability (r = .86) and an internal consistency ranging from .92 for the subscale sexual abuse to .66 for the subscale physical neglect [40–42].

#### Distrust game

**Fig. 1:**
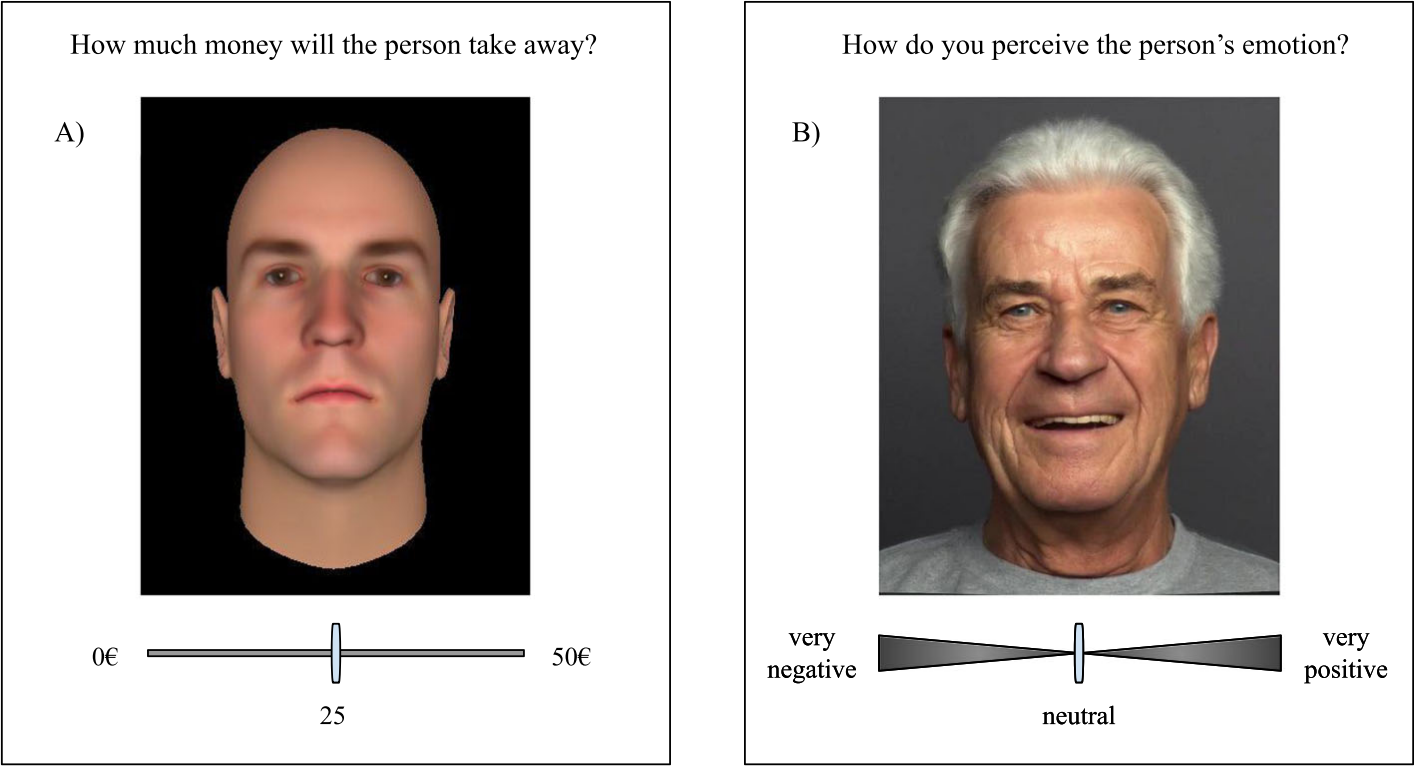
Layout of the distrust game and emotion rating task. Panel A presents an exemplary slide for the distrust game and panel B for the emotion rating task

The distrust game we employed herein was a modified version of a game first introduced by McEvily, Radzevick, and Weber [36] and adapted as a theoretical game by Thielmann and Hilbig [43]. Participants played a hypothetical version of the game (without real monetary stakes), which we instructed as follows: “Imagine you are playing a game with another person. At the beginning of the game, you and your partner each receive a seed capital of 50€. Your partner then has the chance to take any amount of your 50€, without you being able to react. If you had the choice who to play with, it would thus be important to make a good judgement about who would take more or less money from you. Next, you are going to see the faces of different individuals. Your task will be to estimate how much money each person would take away from you. There are no right or wrong answers. We are only interested in your personal judgement. You are going to see two rounds of faces. After the first round, you will receive a feedback about how much money each person would have taken from you in the first round, which you can decide to use to adjust your estimates in round two.” In our adaptation of the distrust game, participants saw a total of 42 faces with 7 different levels of trustworthiness, ranging from very untrustworthy (−3) to very trustworthy (+3). Faces were taken from the stimulus set developed by Todorov and Oosterhof [44], who created computerized faces that systemically vary in their level of facial trustworthiness. We presented stimuli in two rounds, so that participants saw 21 stimuli in each round and each level of trustworthiness 3 times. We presented different identities in each trial and randomized their order. In each trial, participants indicated the amount of money they thought the depicted person would take away from them (0–50 Euros in 1 Euro steps), with high amounts an indicator of distrust toward the interaction partner. After the first round, we presented a positive feedback. We displayed each identity from the first round with the (fictitious) amount of money this person would have taken from them, ranging between 0 and 7 Euros, with the average extraction across participants being 1.2 Euros. Fictitious extractions were assigned randomly to the identities (i.e., were independent of the level of trustworthiness). We also presented the verbal summary “On average, participants in round 1 would have taken less than 2 Euros from you.” Next followed round 2 of the distrust game. Figure 1, panel a, illustrates a distrust game trial.

#### Emotion rating task

In this task, participants saw 60 faces of positive or negative emotional valence and varying intensity from the FACES stimulus set [45]. Positive stimuli displayed varying levels of joy (including zero joy, i.e. neutral faces) and negative stimuli displayed angry or fearful expressions (again including neutral faces). We presented 30 positive and 30 negative faces (15 fearful, 15 angry) in a randomized order. Emotional intensity varied at four levels, ranging from 0 (no emotion present, neutral face) to 4 (fully expressed emotion). Thus, each intensity was displayed five times for each valence (i.e. five positive pictures of intensity 4 were shown, five positive pictures of intensity 3 were shown etc.). Participants rated the valence of the emotional expression for each face, ranging from “very negative” to “very positive”. Participants provided this rating on a slider scale with nine levels and three verbal anchors. The left extreme of the slider was anchored as “very negative” and the right extreme of the slider was anchored as “very positive”, with the middle labelled as “neutral” (see Fig. 1 panel b). Numerically, this scale varied from − 4 to + 4, with − 4 indicating a “very negative” rating and + 4 indicating a “very positive” rating and 0 being the neutral point.

### Data analytic plan

We conducted linear mixed effects models (LMMs) for both the distrust game and the emotion rating task, to account for the repeated measures design and crossed nature of raters and stimuli. We conducted analyses in R, using the lmer function from the *lme4* package [46]. We additionally used the *lmerTest* package [47] to compute *p*-values and the *sjstats* package [48] to compute effect sizes. All data and analysis code in R is provided in an online repository (https://osf.io/pufy2/).

To test whether participants with higher levels of CM show greater levels of distrust overall (H1a) and whether they are  less reactive to positive feedback presented after the first round (H1b), we employed a LMM with random intercepts for participants and stimuli (crossed). The model comprised distrust ratings (amount of money expected to be taken) as the outcome, predicted by the trustworthiness of the stimulus, the CTQ sum score, a dummy predictor coding whether the rating was made in the first round (before the feedback) or in the second round (following the feedback). For the dummy predictor, the first round was coded as 0 (the baseline condition) and the second round was coded as 1. The model further included all two and three-way interactions. Participants’ CTQ scores were centered on the grand mean and trustworthiness was centered such that an average trustworthiness was coded as 0. We modelled random slopes for the predictors trustworthiness and round.

To analyze data from the emotion rating task, we first computed difference scores between picture intensity and participants’ valence ratings, to determine whether the rating was negatively or positively shifted. In detail, we subtracted the stimulus intensity from the rating. For instance, if a stimulus had the intensity + 1 (i.e., it was a mildly positive stimulus), but the participant rated the stimulus as − 1 (mildly negative), their difference score would be − 2, because their rating was negatively shifted by two points. The same applies to positive shifts: If a stimulus with an intensity of + 1 was rated as + 3, this would indicate a positive shift by 2 points. These difference scores served as outcomes in a LMM. The model included a dummy-predictor coding stimulus valence (0 = positive, 1 = negative), grand-mean centered CTQ scores, and their interaction. We again modelled random intercepts for participants and stimuli (crossed), and a random slope for stimulus valence.

## Results

### CM distribution

The CTQ sum scores ranged from 25 to 120, whereby 25 indicates that a participant chose the lowest category on each item (“not at all/never”), and 120 indicates a participant chose the highest category (“very often”) on almost all items. The average CTQ sum score was *M* = 45.05, indicating lower average levels of CM in this sample, though the dispersion was substantial with *SD* = 21.15. Beyond the overall sum score, the CTQ also comprises five subscales for specific types of CM, which are presented in Table 3. The average subscale scores indicated that participants reported higher levels of emotional neglect and abuse than physical neglect, physical abuse, or sexual abuse. The subscales can be further divided into categories of minimal, low, moderate, and severe levels of trauma (see [40] for details), which are also presented in Table 3. These severity thresholds revealed that almost a fourth of the sample indicated severe to extreme levels of emotional abuse, approximately a sixth of all participants reported severe to extreme levels of emotional neglect and sexual abuse, and around one tenth of all participants indicated severe to extreme physical neglect and abuse.

**Table 3:** Means, standard deviations and frequencies for the different severity categories of the CTQ subscales

	*M*	*SD*	*minimal*	*low*	*moderate*	*severe*
Emotional neglect	11.22	5.70	278 (50.6%)	122 (22.2%)	56 (10.2%)	93 (16.9%)
Emotional abuse	11.28	6.18	259 (47.2%)	104 (18.9%)	52 (9.5%)	134 (24.4%)
Physical neglect	7.94	3.57	316 (57.6%)	93 (16.9%)	79 (14.4%)	61 (11.1%)
Physical abuse	6.97	3.69	423 (77.1%)	41 (7.5%)	35 (6.4%)	50 (9.1%)
Sexual abuse	7.63	5.41	388 (70.7%)	31 (5.7%)	51 (9.3%)	79 (14.4%)

*Note.* Thresholds for the subscales were based on [40]

### Distrust game

**Fig. 2:**
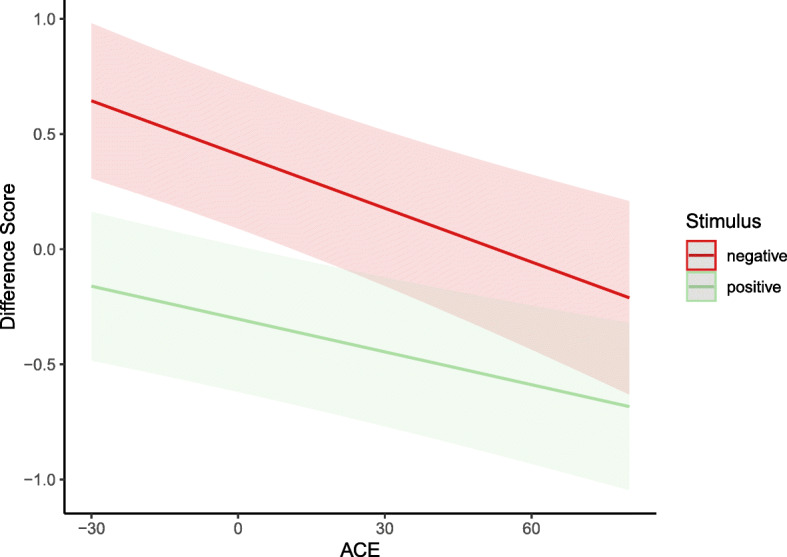
Model plot for the linear mixed-effect model testing hypothesis 1. The grand-mean centered level of childhood maltreatment (CM, measured using the childhood trauma questionnaire, [40]) is presented on the x-axis, and the level of distrust (indicated via monetary estimates in the distrust game) is presented on the y-axis. Regression lines are plotted separately for the first and second round

Results are presented in Table 4. Supporting H1a, the main effect of the CTQ was significant and positive, indicating that higher levels of CM entailed higher levels of distrust. Moreover, the main effect of round was significant and negative, indicating that participants overall showed lower levels of distrust in the second round (following the positive feedback they were presented with after completing the first round). As expected, the significant positive interaction between round and the CTQ indicated that the decrease in distrust from the first to the second round was weaker in individuals with higher levels of CM. Figure 2 illustrates this interaction. Moreover, the main effect of stimulus trustworthiness was significant and negative, indicating that participants provided lower distrust ratings when stimulus trustworthiness increased. This finding was unrelated to our hypotheses but functioned as a manipulation check, indicating that the stimulus material had the intended effect. Also unrelated to our pre-registered hypotheses (and therefore to be considered an exploratory finding), we observed a significant negative two-way interaction between stimulus trustworthiness and CTQ score, which indicated that the effect of stimulus trustworthiness was stronger at lower CTQ levels. Lastly, we observed a significant three-way interaction between CTQ, stimulus trustworthiness, and round, which was also not part of our hypotheses.

**Table 4:** Results from a linear mixed effects model predicting distrust ratings with trustworthiness of the stimuli, round, amount of CM, and their interactions

			*Distrust*	
	*Est.*	*β*	*SE*	*p*
Intercept	24.92		0.73	<.001
Trustworthiness	**−2.32**	**−0.29**	**0.32**	**<.001**
Round	**−11.43**	**−0.36**	**1.00**	**<.001**
CM	**0.08**	**0.10**	**0.02**	**<.001**
Trustworthiness × Round	0.79	0.07	0.45	.087
Trustworthiness × CM	**−0.01**	**−0.02**	**0.00**	**.007**
Round × CM	**0.06**	**0.06**	**0.02**	**.004**
Trustworthiness × CM × Round	**−0.01**	**− 0.01**	**0.00**	**.030**

*Note*: *Est*. = Estimate, *CM* = level of childhood maltreatment measured using the Childhood Trauma Questionnaire. Round was coded as round one = 0 and round two = 1. Significant effects are highlighted in boldface (*p* < .05)

Following the suggestion by an anonymous reviewer, we repeated the analysis for the CTQ subscales emotional neglect, emotional abuse, physical neglect, physical abuse, and sexual abuse to determine whether one specific type of CM was driving the effects. All CTQ subscales individually showed the same association with distrust ratings as the CTQ total score (see Tables S1, S2, S3, S4 and S5 in the online supplement). All subscales were also substantially correlated with each other (see Table S7). Thus, using them as a set of predictors for the distrust ratings introduced issues of multicollinearity and rendered the interpretation of results difficult. In the model using all subscales as simultaneous predictors, none of the subscales were consistently associated with distrust ratings and only some isolated interaction terms reached significance (see Table S6). Following a second suggestion by the reviewer, we also repeated the analysis with the CTQ total score adjusting for income and education level as a proxy for socioeconomic status. Using these covariates, we observed the same pattern of results as without them (see Table S14).

### Emotion rating task

**Fig. 3:**
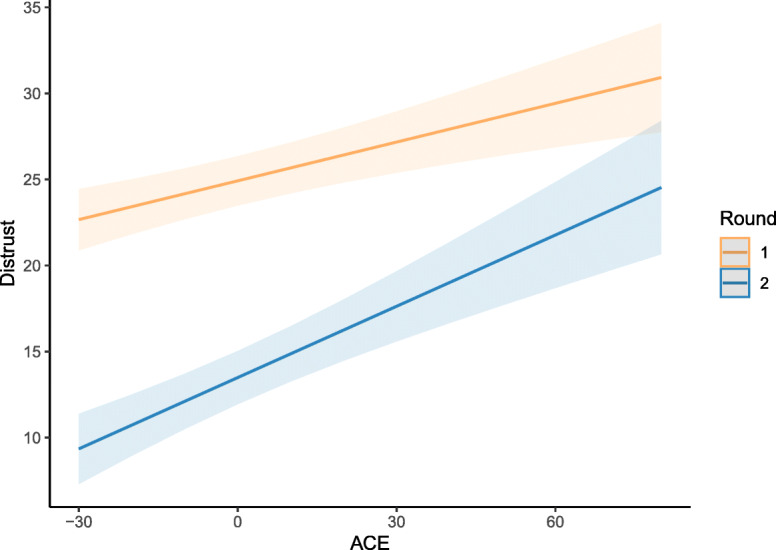
Model plot for the linear mixed-effect model testing hypothesis 2. The grand-mean centered level of childhood maltreatment (CM, measured using the childhood trauma questionnaire, [40]) is presented on the x-axis, and the difference score that served as outcome in the emotion rating task (emotion rating- stimulus intensity) is presented on the y-axis. Regression lines are plotted separately for negative and positive stimuli

Results are presented in Table 5. We observed a significant, negative main effect of the CTQ sum score, indicating that individuals with higher levels of CM tended to show more negatively shifted emotion ratings, which supported hypothesis 2. We did not observe an interaction between the CTQ score and stimulus type (positive vs. negative). Figure 3 presents the model plot for the association between the CTQ score and the emotion rating. Again, we repeated the analysis for each CTQ subscale and all scales showed the same pattern of association with emotion ratings as the CTQ total score (see Table S8, S9, S10, S11 and S12). As for the distrust game, entering all subscales together in one model introduced problems with multicollinearity and results must be interpreted with caution. Here, the subscales emotional abuse and sexual abuse showed significant associations with emotion ratings (see Table S13). Additionally, the results also replicated when adjusting the model using the CTQ total score for education and income level (Table S15).

**Table 5:** Results from a linear mixed effects model predicting the emotion rating (difference score) with stimulus valence, level of CM, and their interaction

	*Emotion Rating*			
	*Est.*	*β*	*SE*	*p*
Intercept	0.41		0.16	.015
Valence	**−0.71**	**− 0.22**	**0.23**	**.003**
CM	**−0.01**	**−0.10**	**0.00**	**<.001**
Valence × CM	0.00	0.03	0.00	.162

*Note*: *Est.* = Estimate, *CM* = level of childhood maltreatment measured using the Childhood Trauma Questionnaire. Valence was coded as negative (angry or fearful) stimulus = 0, positive (happy) stimulus = 1. Significant effects are highlighted in boldface (*p* < .05)

## Discussion

The present study investigated whether a history of CM entails cognitive alterations similar to those proposed in cognitive models of PTSD [8, 9]. Specifically, we tested components of the model proposing that trauma entails increased distrust and perceived interpersonal threat (i.e., that traumatized individuals are less trusting and tend to perceive others as more threatening) in 549 participants who self-reported their level of CM. We assessed the association between CM and participants’ performance in a hypothetical distrust game and an emotion rating task that contained negatively valenced faces signaling threat. Results supported the central hypotheses that individuals with higher levels of CM show more distrust and negatively shifted emotion ratings. In other words, in addition to traumatic events that might culminate in PTSD symptomatology, all trauma occurring during childhood, including trauma types that would not qualify as a traumatic event based on criterion A of the DSM-5 PTSD diagnosis [10], may have far-reaching consequences and entail greater distrust and threat perception later in life.

In detail, we found that individuals with more CM rated interaction partners as less trustworthy in the distrust game (expecting them to take away more of their money overall). This finding is in line with previous findings on higher self-reported levels of distrust in individuals with CM of varying age groups [14–17]. It contrasts a study that found no difference between girls with and without a history of physical or sexual abuse in the trust game [18]. It is possible that this discrepancy reflects the usefulness of assessing CM continuously rather than using between-groups approaches that set somewhat arbitrary thresholds of presence vs. absence of CM. Moreover, the present sample covered a range of different types of CM rather than focusing on a specific type of event.

As a second component in the distrust game, we implemented a feedback condition during which we told participants that interaction partners in the first round took very little money from them. We hypothesized that participants with a higher level of CM would be less responsive to this positive feedback. Results showed that participants generally did adapt their ratings in round 2, but that those with more ACE did so to a lesser degree, supporting our hypothesis. This finding adds to evidence that adolescent girls with ACE show a lower ability than non-exposed girls to learn which individuals are trustworthy in the trust game [19]. In a different study, participants with PTSD showed stronger reductions in their investments when an interaction partner switched from cooperative to uncooperative behavior than HCs, which suggests that learning from negative feedback (i.e., someone is untrustworthy) may be intact [21]. While preliminary, taken together, these findings suggest that individuals with CM may struggle to encode positive feedback in the sense of ‘person X was *not* untrustworthy after all’, but are able to adapt to someone revealing them self as untrustworthy. Thus, distrust may be somewhat resistant to corrective feedback.

In the second paradigm, we tested whether individuals with higher levels of CM are more likely to perceive faces as negative and potentially threatening. Results showed that individuals with a higher level of CM tended to perceive angry, fearful, and happy faces as more negative. By creating a difference score subtracting the actual intensity score of the stimulus from the rating, we were able to create an outcome representing positive or negative shifts in the ratings, or even bias. Overall, participants with more CM tended to show stronger negative shifts, that is, they perceived stimuli more negatively than they actually were. This adds to the existing literature of emotion processing in individuals with CM and extends the age range of samples to adults (as outlined in the introduction, most previous work focused on children). It is even possible that some of the previous studies that detected a higher emotion recognition accuracy or sensitivity for negative emotions are reflective of the same bias we discovered herein [25–28, 30]. The present results also parallel two studies which observed that children with CM had a tendency to interpret ambiguous situations and faces as angry [23, 24], and two studies which demonstrated that adults with CM interpret neutral expressions as more angry, contemptuous, or fearful [31, 32]. It is possible this reflects individual learning histories of individuals with CM, such that they needed to detect threatening cues (e.g., an angry-looking parent) early to avoid negative consequences. In such an environment, a ‘false alarm’, that is, falsely identifying a face as angry when it is not, would be less consequential than a ‘miss’ of not noticing the angry person. In this context, an increased sensitivity to, or even bias for, perceiving threat would been adaptive, whereas later in life and with changing contexts it loses this adaptive component.

### Limitations and research implications

The present study had a number of limitations, many of which hold direct implications for future research. First, there are limitations pertaining to the sample we recruited. In order to recruit a large sample with varying levels of CM, we conducted the study online. While we took care to advertise the study through a broad range of channels, including those directly related to CM, participants in the sample tended to be on the lower end of the CM spectrum. Yet, a subgroup of participants also endorsed severe to extreme levels of all forms of CM, but the distribution remained heavily skewed. Arguably, this may well reflect the distribution of CM in the general publication, since more extreme levels of CM are rarer [35], but it also led to a relatively low endorsement of certain types of CM, specifically physical neglect and abuse. Yet, when looking at the individual CTQ scales as predictors of distrust and emotion ratings, we observed the same patterns as for the total score (see supplement). Future studies should consider directly targeting their recruitment strategy toward individuals with different types of CM and assessing CM subtypes in detail, for instance by employing interviews (e.g. [49]) in addition to self-report scales.

Second, due to the online nature of the study, participants did not complete structured clinical interviews but only self-reported whether they had previously received any diagnoses from a mental health professional. This entails the limitation that we cannot reliably say how many participants in the study may have had a formal diagnosis of a trauma-related disorder, especially PTSD. Beyond providing the general picture that the sample was somewhat affected by psychopathology, we did not deem these self-reports reliable enough to include them in our analyses and draw any valid conclusions from. We cannot exclude that the self-reported diagnoses may have contained instances of ‘self-diagnosis’, as well as diagnoses that were made by a general practitioner or other health care provider that would not be confirmed based on standardized assessments. However, since we did not formulate hypotheses based on diagnostic status but rather considered CM severity dimensionally, this did not directly affect any of the hypotheses we tested.

This is not to say that assessing potential differences based on presence or absence of PTSD or other conditions would not be interesting and important. We assume that many of the effects we observed would be potentiated in those with PTSD, especially a further restriction of their responsiveness to positive feedback in the distrust game. Considering the range of disorders that are associated with a history CM, disturbances in threat-processing and trust likely also have implications for various conditions beyond PTSD. Indeed, previous research suggests that altered threat-processing and trust are also present in disorders such as depression [50] or BPD [51]. Individuals with BPD, in particular, are affected by substantial levels of CM [39] and show reduced trust in the trust game [52] and a tendency to perceive neutral or ambivalent faces as threatening [53]. Both of these processes have also been discussed as potential contributors to the marked interpersonal dysfunction in BPD [54]. Future research could address whether the level of CM that individuals with depression or BPD experienced explains the magnitude of alterations in trust and threat perception. Moreover, both processes also have the potential to help explain the marked disturbances in interpersonal relationships that individuals with complex PTSD report [55]. In order to reliably test this, future studies will need to include a standardized assessment of the conditions of interest.

A third limitation pertaining to the sample is that it is restricted with regard to gender, age, and ethnicity. The current results are largely based on young, well-educated, cis-gender women without a migration background, and this restricts the generalizability of the results. Beyond increasing generalizability, it is particularly important to consider sampling individuals from diverse genders, ethnicities, and sexual orientations, because CM and CM-related negative outcomes are more prevalent in the broader LGBTQ+ community, in transgender people, and people of color [56–59]. Moreover, it would be highly relevant to assess whether the results we observed also replicate in samples of older adults. The present sample was rather young, thus it is possible that the associations between distrust, emotion processing, and CM were inflated by the relative recency of CM. In other words, the association between CM and any outcome can be expected to be larger in a sample that is on average 25 years old (thus having self-reported CM events that may have happened as recently as 8 years ago), versus in a sample that is on average 50 years old. This also points to a need to more closely assess the timing of CM, that is, at what age adverse events took place.

A second group of limitations pertains to the paradigms we employed; the distrust game and the emotion rating task. A main limitation with regard to the distrust game is that it was a hypothetical game. That is, participants did not receive real money and were not actually paired with an interaction partner, but only told to imagine that they were playing this game. While previous studies have shown that hypothetical adaptations of economic games are generally feasible and reliable [60, 61], others have argued that participants behave substantially differently when real incentives are involved [62, 63]. Moreover, naturally unfolding interaction effects between actual pairs of players cannot be assessed in hypothetical settings. Real incentives and interaction partners were not possible in the online setting, but not having them also allowed for greater experimental control, because all participants saw the same avatars and their (fictitious) extractions. Nonetheless, it will be important for future studies to replicate the results using real incentives and interaction partners in laboratory settings.

With regard to the emotion rating task, we were also limited by feasibility constraints, aiming to keep the study at a manageable length that would not be too burdensome for participants and lead to a high number of drop-out or low participation rate. For the emotion rating task, this meant restricting the number of trials we could present. We therefore restricted the number of specific affects we included and only compared positive (happy) versus negative (angry or fearful) faces. In a design that allows more trials (e.g. because participants complete the study in a controlled laboratory setting and are compensated for their participation), assessing more specific types of negative affect, as well as ambivalent or neutral expressions, would be desirable.

Lastly, our study was limited by the fact that it was only of a cross-sectional nature. As alluded to above, the effects of CM may play out differently depending on the relative recency of CM, but they may also play out differently within the same person on a day-to day basis. It will be important to assess whether increased distrust and threat sensitivity are relatively stable across situations or whether they fluctuate based on context factors such as negative affect, interaction partners, or triggers. For instance, it is possible that a participant may be largely unaffected by distrust in moments when they are in a positive mood, surrounded by close friends, and when they have had a day without any reminders of their trauma, but the picture may shift entirely when they are stressed, surrounded by strangers, or on the morning following a nightmare. Thus, we would argue that a combination of the herein presented cross-sectional, between-person perspective with data on within-person fluctuations might ultimately be the most well suited to further investigate social-cognitive process in those with CM.

### Clinical implications

Given further replication, the present study also holds a number of potential clinical implications. First, the results suggest that individuals with CM might be less trustful and more prone to interpreting facial expressions as negative. This has implications for forming therapeutic relationships and it could affect treatment progress and outcomes, if overlooked. For instance, the results suggest that therapists can expect patients with CM to be somewhat distrusting at the start of the therapeutic process and to interpret the therapist’s facial expressions more readily as negative. While this problem could be addressed within the therapeutic session, it is likely something that also tends to play out regularly in the patient’s daily life and that may warrant further intervention. Specific interventions suited to address it depend heavily on the therapeutic method and could range from psychoeducation, cognitive reappraisal (and seeking out real-life situations to practice this in) to schema modification.

Moreover, the current results suggest that it is important to consider CM as a factor that entails negative outcomes, even if it does not result in PTSD or other trauma-related disorders. Even types and intensities of CM that are not universally recognized as trauma in a narrow sense were associated with increased distrust and threat sensitivity in the present study. Thus, taking a detailed biographical history that includes a specific assessment of all types of CM could be beneficial for selecting treatment strategies and creating a therapeutic relationship, irrespective of what the main diagnosis is.

Importantly, we want to emphasize that all participants reacted substantially to the positive feedback presented after the first round of the distrust game. On average, participants adjusted their expectations of the amount of money that would be taken from them by around 11.5 Euros. Even though those with higher CM levels adapted their expectation significantly less, they still did react to the positive feedback. This suggests that correcting experiences made in real life, which can be created and encouraged in the therapeutic process, are likely fruitful, even if distrust and threat perception are somewhat change resistant at first.

## Conclusions

The study demonstrated that CM is associated with increased distrust and decreased trust-learning (in a distrust game) and increased interpersonal threat perception (in a facial affect rating task). This suggests that cognitive alterations of increased distrust and perceived threat, that are theorized to be central to the development and maintenance of PTSD [8, 9], are also present in individuals with varying types and levels of CM, including those that would not meet diagnostic thresholds for traumatic events based on diagnostic systems. Rather, the present results suggested the association between traumatic load and distrust/threat perception may follow a dose-response relationship. Given replication, this implies that individuals with varying types and levels of CM may similarly benefit from cognitive interventions designed for PTSD.

## Supplementary Information

**Additional file 1.**

## Data Availability

Data and analysis code for this study are freely available and can be accessed in the following OSF online repository: https://osf.io/pufy2/

## Footnotes

1 Data was collected in April of 2020, during which there was a partial lockdown in Germany due to the Covid-19 pandemic; hence in-person data collection was not possible.
