# Supplementary material for: Childhood maltreatment is associated with distrust and negatively biased emotion processing
Source: Borderline Personal Disord Emot Dysregul. 2021 Feb 3;8:5. doi: 10.1186/s40479-020-00143-5 (PMC7856450; doi:10.1186/s40479-020-00143-5)
Supplement: Supplementary file 1 — Additional file 1. [file 40479_2020_143_MOESM1_ESM.docx]

**Supplemental Materials**

**for**

**Childhood maltreatment is associated with distrust and negatively biased emotion processing**

Johanna Hepp, Sara E. Schmitz, Jana Urbild, Kathrin Zauner, Inga Niedtfeld

Correspondence should be addressed to: Johanna Hepp, Department of Psychosomatic Medicine and Psychotherapy, Central Institute of Mental Health, J5, 68159 Mannheim, Germany. Email: Johanna.Hepp@zi-mannheim.de. Phone: +49 621 1703 4427

**Supplemental analyses with the CTQ subscales**

To determine whether the effects we observed for the CTQ total score were driven by one particular type of childhood maltreatment, we repeated the analyses using the CTQ subscales. The subscales included emotional neglect, emotional abuse, physical neglect, physical abuse, and sexual abuse.

*Distrust Game*

For the distrust game, all results replicated when using the individual CTQ subscales instead of the CTQ total score (see Tables S1- S5). This suggested that all types of childhood maltreatment were associated with distrust. When including all CTQ subscales simultaneously, none of them reached statistical significance (Table S6). We would argue that was likely due to the substantial multicollinearity including all subscales simultaneously incurred. As depicted in Table S7, the subscales were all very highly correlated (all above .55).

Table S1. *Results from a linear mixed effects model predicting distrust ratings with trustworthiness of the stimulus, round, level of emotional neglect, and their interactions.*

|  | *Distrust* | | | |
| --- | --- | --- | --- | --- |
|  | *Est.* | *β* | *SE* | *p* |
| Intercept | 21.97 |  | 1.05 | <.001 |
| Trustworthiness | **-2.03** | **-0.29** | **0.35** | **<.001** |
| Round | **-13.95** | **-0.72** | **1.34** | **<.001** |
| Emotional Neglect | **0.26** | **0.09** | **0.07** | **<.001** |
| Trustworthiness × Round | **1.06** | **0.10** | **0.46** | **.028** |
| Trustworthiness × Emotional Neglect | **-0.03** | **-0.02** | **0.01** | **.046** |
| Round × Emotional Neglect | **0.23** | **0.08** | **0.08** | **.005** |
| Trustworthiness × Emotional Neglect × Round | **-0.02** | **-0.02** | **0.01** | **.026** |

*Note*: Est. = Estimate, emotional neglect measured using the Childhood Trauma Questionnaire. Round was coded as round one = 0 and round two = 1. Significant effects are highlighted in boldface (*p* < .05).

Table S2. *Results from a linear mixed effects model predicting distrust ratings with trustworthiness of the stimuli, round, level of emotional abuse, and their interactions.*

|  | *Distrust* | | | |
| --- | --- | --- | --- | --- |
|  | *Est.* | *β* | *SE* | *p* |
| Intercept | 22.24 |  | 1.01 | <.001 |
| Trustworthiness | **-1.98** | **-0.29** | **0.35** | **<.001** |
| Round | **-13.93** | **-0.72** | **1.30** | **<.001** |
| Emotional Abuse | **0.24** | **0.09** | **0.06** | **<.001** |
| Trustworthiness × Round | **1.00** | **0.10** | **0.46** | **.035** |
| Trustworthiness × Emotional Abuse | **-0.03** | **-0.02** | **0.01** | **.013** |
| Round × Emotional Abuse | **0.22** | **0.09** | **0.07** | **.003** |
| Trustworthiness × Emotional Abuse × Round | -0.02 | -0.01 | 0.01 | .056 |

*Note*: Est. = Estimate, emotional abuse measured using the Childhood Trauma Questionnaire. Round was coded as round one = 0 and round two = 1. Significant effects are highlighted in boldface (*p* < .05).

Table S3. *Results from a linear mixed effects model predicting distrust ratings with trustworthiness of the stimuli, round, level of physical neglect, and their interactions.*

|  | *Distrust* | | | |
| --- | --- | --- | --- | --- |
|  | *Est.* | *β* | *SE* | *p* |
| Intercept | 22.48 |  | 1.12 | <.001 |
| Trustworthiness | **-1.80** | **-0.29** | **0.36** | **<.001** |
| Round | **-13.68** | **-0.72** | **1.42** | **<.001** |
| Physical Neglect | **0.31** | **0.07** | **0.11** | **.004** |
| Trustworthiness × Round | 0.83 | 0.10 | 0.47 | .083 |
| Trustworthiness × Physical Neglect | **-0.07** | **-0.03** | **0.02** | **.001** |
| Round × Physical Neglect | **0.28** | **0.06** | **0.13** | **.027** |
| Trustworthiness × Physical Neglect × Round | -0.01 | 0.00 | 0.02 | .747 |

*Note*: Est. = Estimate, physical neglect measured using the Childhood Trauma Questionnaire. Round was coded as round one = 0 and round two = 1. Significant effects are highlighted in boldface (*p* < .05).

Table S4. *Results from a linear mixed effects model predicting distrust ratings with trustworthiness of the stimuli, round, level of physical abuse, and their interactions.*

|  | *Distrust* | | | |
| --- | --- | --- | --- | --- |
|  | *Est.* | *ß* | *SE* | *p* |
| Intercept | 22.33 |  | 1.03 | <.001 |
| Trustworthiness | **-2.11** | **-0.29** | **0.35** | **<.001** |
| Round | **-13.34** | **-0.72** | **1.31** | **<.001** |
| Physical Abuse | **0.37** | **0.09** | **0.10** | **<.001** |
| Trustworthiness × Round | **1.03** | **0.10** | **0.46** | **.031** |
| Trustworthiness × Physical Abuse | -0.03 | -0.01 | 0.02 | .143 |
| Round × Physical Abuse | **0.27** | **0.06** | **0.12** | **.027** |
| Trustworthiness × Physical Abuse × Round | **-0.03** | **-0.02** | **0.02** | **.038** |

*Note*: Est. = Estimate, physical abuse measured using the Childhood Trauma Questionnaire. Round was coded as round one = 0 and round two = 1. Significant effects are highlighted in boldface (*p* < .05).

Table S5. *Results from a linear mixed effects model predicting distrust ratings with trustworthiness of the stimuli, round, level of sexual abuse, and their interactions.*

|  | *Distrust* | | | |
| --- | --- | --- | --- | --- |
|  | *Est.* | *β* | *SE* | *p* |
| Intercept | 23.12 |  | 0.91 | <.001 |
| Trustworthiness | **-2.07** | **-0.29** | **0.34** | **<.001** |
| Round | **-12.76** | **-0.72** | **1.19** | **<.001** |
| Sexual Abuse | **0.24** | **0.08** | **0.07** | **<.001** |
| Trustworthiness × Round | **0.99** | **0.10** | **0.46** | **.036** |
| Trustworthiness × Sexual Abuse | **-0.03** | **-0.02** | **0.01** | **.017** |
| Round × Sexual Abuse | **0.17** | **0.06** | **0.08** | **.040** |
| Trustworthiness × Sexual Abuse × Round | **-0.03** | **-0.02** | **0.01** | **.021** |

*Note*: Est. = Estimate, sexual abuse measured using the Childhood Trauma Questionnaire. Round was coded as round one = 0 and round two = 1. Significant effects are highlighted in boldface (*p* < .05).

Table S6. *Results from a linear mixed effects model predicting distrust ratings with trustworthiness of the stimuli, round, level of CTQ subscales, and their interactions.*

|  | *Distrust* | | | |
| --- | --- | --- | --- | --- |
|  | *Est.* | *β* | *SE* | *p* |
| Intercept | 21.86 |  | 1.14 | <.001 |
| Trustworthiness | **-1.86** | **-0.29** | **0.36** | **<.001** |
| Round | **-14.03** | **-0.72** | **1.45** | **<.001** |
| Emotional Abuse | 0.07 | 0.03 | 0.13 | .571 |
| Physical Abuse | 0.16 | 0.04 | 0.16 | .300 |
| Sexual Abuse | 0.10 | 0.03 | 0.09 | .275 |
| Emotional Neglect | 0.15 | 0.05 | 0.14 | .302 |
| Physical Neglect | -0.17 | -0.04 | 0.17 | .356 |
| Trustworthiness × Round | **0.95** | **0.10** | **0.47** | **.049** |
| Trustworthiness × Emotional Abuse | -0.03 | -0.02 | 0.02 | .277 |
| Trustworthiness × Physical Abuse | 0.04 | 0.02 | 0.02 | .226 |
| Trustworthiness × Sexual Abuse | -0.02 | -0.01 | 0.02 | .335 |
| Trustworthiness × Emotional Neglect | 0.03 | 0.02 | 0.03 | .261 |
| Trustworthiness × Physical Neglect | **-0.08** | **-0.04** | **0.03** | **.023** |
| Round × Emotional Abuse | 0.16 | 0.06 | 0.16 | .302 |
| Round × Physical Abuse | 0.02 | 0.00 | 0.19 | .920 |
| Round × Sexual Abuse | 0.04 | 0.01 | 0.11 | .730 |
| Round × Emotional Neglect | 0.07 | 0.02 | 0.17 | .686 |
| Round × Physical Neglect | -0.05 | -0.01 | 0.21 | .804 |
| Trustworthiness × Emotional Abuse × Round | -0.00 | -0.00 | 0.02 | .955 |
| Trustworthiness × Physical Abuse × Round | -0.03 | -0.02 | 0.03 | .198 |
| Trustworthiness × Sexual Abuse × Round | -0.02 | -0.02 | 0.01 | .123 |
| Trustworthiness × Emotional Neglect × Round | -0.03 | -0.02 | 0.02 | .147 |
| Trustworthiness × Physical Neglect × Round | **0.08** | **0.04** | **0.03** | **.006** |

*Note*: Est. = Estimate, Subscales of Childhood Trauma Questionnaire. Round was coded as round one = 0 and round two = 1. Significant effects are highlighted in boldface (*p* < .05).

Table S7. *Correlation table for the CTQ subscales.*

|  | *Emo neglect* | *Emo abuse* | *Phys neglect* | *Phys abuse* | *Sexual abuse* |
| --- | --- | --- | --- | --- | --- |
| *Emo neglect* |  |  |  |  |  |
| *Emo abuse* | 0.86 |  |  |  |  |
| *Phys neglect* | 0.75 | 0.74 |  |  |  |
| *Phys abuse* | 0.66 | 0.69 | 0.68 |  |  |
| *Sexual abuse* | 0.59 | 0.57 | 0.58 | 0.55 |  |

*Emotion Rating Task*

As for the distrust game, we also repeated the analysis for the emotion rating task with each CTQ subscale instead of the CTQ total score. Again, results replicated when considering each CTQ subscale individually (Tables S7- S12). When including all subscales as simultaneous predictors, only emotional abuse and sexual abuse emerged as significant. However, as we argued above, including all subscales simultaneously is problematic due to high collinearity of the subscales, which gravely limits the interpretability of the this model (Table S13).

Table S8. *Results from a linear mixed effects model predicting the emotion rating (difference score) with the valence of the stimulus, level of emotional neglect, and their interaction.*

|  | *Emotion Rating* | | | |
| --- | --- | --- | --- | --- |
|  | *Est.* | *β* | *SE* | *p* |
| Intercept | 0.67 |  | 0.18 | <.001 |
| Emotional Neglect | **-0.02** | **-0.08** | **0.01** | **<.001** |
| Valence | **-0.81** | **-0.44** | **0.25** | **.002** |
| Emotional Neglect x Valence | 0.01 | 0.03 | 0.01 | .299 |

*Note*: Est. = Estimate, emotional neglect measured using the Childhood Trauma Questionnaire. Valence was coded as negative (angry or fearful) stimulus = 0, positive (happy) stimulus = 1. Significant effects are highlighted in boldface (*p* < .05).

Table S9. *Results from a linear mixed effects model predicting the emotion rating (difference score) with the valence of the stimulus, level of emotional abuse, and their interaction.*

|  | *Emotion Rating* | | | |
| --- | --- | --- | --- | --- |
|  | *Est.* | *β* | *SE* | *p* |
| Intercept | 0.75 |  | 0.18 | <.001 |
| Emotional Abuse | **-0.03** | **-0.11** | **0.01** | **<.001** |
| Valence | **-0.87** | **-0.44** | **0.24** | **<.001** |
| Emotional Abuse x Valence | 0.01 | 0.05 | 0.01 | .071 |

*Note*: Est. = Estimate, emotional abuse measured using the Childhood Trauma Questionnaire. Valence was coded as negative (angry or fearful) stimulus = 0, positive (happy) stimulus = 1. Significant effects are highlighted in boldface (*p* < .05).

Table S10. *Results from a linear mixed effects model predicting the emotion rating (difference score) with the valence of the stimulus, level of physical neglect, and their interaction.*

|  | *Emotion Rating* | | | |
| --- | --- | --- | --- | --- |
|  | *Est.* | *β* | *SE* | *p* |
| Intercept | 0.65 |  | 0.18 | <.001 |
| Physical Neglect | **-0.03** | **-0.07** | **0.01** | **.003** |
| Valence | **-0.75** | **-0.44** | **0.25** | **.004** |
| Physical Neglect x Valence | 0.00 | 0.01 | 0.01 | .753 |

*Note*: Est. = Estimate, physical neglect measured using the Childhood Trauma Questionnaire. Valence was coded as negative (angry or fearful) stimulus = 0, positive (happy) stimulus = 1. Significant effects are highlighted in boldface (*p* < .05).

Table S11. *Results from a linear mixed effects model predicting the emotion rating (difference score) with the valence of the stimulus, level of physical abuse, and their interaction.*

|  | *Emotion Rating* | | | |
| --- | --- | --- | --- | --- |
|  | *Est.* | *β* | *SE* | *p* |
| Intercept | 0.56 |  | 0.18 | .001 |
| Physical Abuse | **-0.03** | **-0.06** | **0.01** | **.010** |
| Valence | **-0.74** | **-0.44** | **0.25** | **.003** |
| Physical Abuse × Valence | 0.00 | 0.01 | 0.01 | .727 |

*Note*: Est. = Estimate, physical abuse measured using the Childhood Trauma Questionnaire. Valence was coded as negative (angry or fearful) stimulus = 0, positive (happy) stimulus = 1. Significant effects are highlighted in boldface (*p* < .05).

Table S12. *Results from a linear mixed effects model predicting the emotion rating (difference score) with the valence of the stimulus, level of sexual abuse, and their interaction.*

|  | *Emotion Rating* | | | |
| --- | --- | --- | --- | --- |
|  | *Est.* | *β* | *SE* | *p* |
| Intercept | 0.63 |  | 0.17 | <.001 |
| Sexual Abuse | **-0.03** | **-0.10** | **0.01** | **<.001** |
| Valence | **-0.83** | **-0.44** | **0.24** | **<.001** |
| Sexual Abuse × Valence | 0.02 | 0.05 | 0.01 | .062 |

*Note*: Est. = Estimate, sexual abuse measured using the Childhood Trauma Questionnaire. Valence was coded as negative (angry or fearful) stimulus = 0, positive (happy) stimulus = 1. Significant effects are highlighted in boldface (*p* < .05).

Table S13. *Results from a linear mixed effects model predicting the emotion rating (difference score) with the valence of the stimulus, level of CTQ subscales, and their interaction.*

|  | *Emotion Rating* | | | |
| --- | --- | --- | --- | --- |
|  | *Est.* | *β* | *SE* | *p* |
| Intercept | 0.66 |  | 0.18 | <.001 |
| Emotional Abuse | **-0.05** | **-0.18** | **0.01** | **<.001** |
| Physical Abuse | 0.02 | 0.04 | 0.01 | .205 |
| Sexual Abuse | **-0.02** | **-0.07** | **0.01** | **.013** |
| Emotional Neglect | 0.02 | 0.06 | 0.01 | .182 |
| Physical Neglect | 0.01 | 0.03 | 0.02 | .403 |
| Valence | **-0.74** | **-0.44** | **0.25** | **.004** |
| Emotional Abuse × Valence | **0.03** | **0.13** | **0.02** | **.035** |
| Physical Abuse × Valence | -0.02 | -0.05 | 0.02 | .278 |
| Sexual Abuse × Valence | 0.02 | 0.07 | 0.01 | .068 |
| Emotional Neglect × Valence | -0.01 | -0.04 | 0.02 | .460 |
| Physical Neglect × Valence | -0.03 | -0.06 | 0.02 | .206 |

*Note*: Est. = Estimate, Subscales of Childhood Trauma Questionnaire. Valence was coded as negative (angry or fearful) stimulus = 0, positive (happy) stimulus = 1. Significant effects are highlighted in boldface (*p* < .05).

**Supplemental analyses adjusting for socioeconomic status**

To assess whether the observed results would replicate after statistically adjusting for participants’ socioeconomic status, we included monthly income and education level as covariates. This seemed particularly relevant in the case of the distrust game as distrust is operationalized via monetary deductions. Education level was not significantly associated with either distrust or emotion ratings. Income, in contrast, was positively associated with both. This indicated that individuals with a higher monthly income expected higher monetary deductions in the distrust game (i.e. showed more distrust) and provided more negative emotion ratings. The detailed results are presented in Tables S14 and S15.

Table S14. *Results from a linear mixed effects model predicting distrust ratings with trustworthiness of the stimuli, round, level of child maltreatment, and their interactions controlled for income and level of education.*

|  | *Distrust* | | | |
| --- | --- | --- | --- | --- |
|  | *Est.* | *β* | *SE* | *p* |
| Intercept | 24.35 |  | 1.91 | <.001 |
| Trustworthiness | **-2.32** | **-0.29** | **0.32** | **<.001** |
| Round | **-11.61** | **-0.73** | **0.99** | **<.001** |
| CM | **0.07** | **0.09** | **0.02** | **<.001** |
| Income | **0.67** | **0.07** | **0.24** | **.006** |
| Level of Education | -0.33 | -0.02 | 0.48 | .500 |
| Trustworthiness × Round | 0.84 | 0.11 | 0.44 | .064 |
| Trustworthiness × CM | **-0.01** | **-0.02** | **0.00** | **.025** |
| Round × CM | **0.06** | **0.08** | **0.02** | **.011** |
| Trustworthiness × CM × Round | -0.01 | -0.02 | 0.00 | .058 |

*Note*: Est. = Estimate, CM = Child Maltreatment measured using the Childhood Trauma Questionnaire. Round was coded as round one = 0 and round two = 1. Significant effects are highlighted in boldface (*p* < .05).

Table S15. *Results from a linear mixed effects model predicting the emotion rating (difference score) with the valence of the stimulus, level of child maltreatment, and their interaction controlled for income and level of education.*

|  | *Emotion Rating* | | | |
| --- | --- | --- | --- | --- |
|  | *Est.* | *β* | *SE* | *p* |
| Intercept | 0.44 |  | 0.19 | .023 |
| CM | **-0.01** | **-0.09** | **0.00** | **<.001** |
| Valence | **-0.70** | **-0.43** | **0.23** | **.003** |
| Income | **0.03** | **0.03** | **0.01** | **.029** |
| Level of Education | -0.03 | -0.01 | 0.03 | .295 |
| CM × Valence | 0.00 | 0.03 | 0.00 | .340 |

*Note*: Est. = Estimate, CM = child maltreatment measured using the Childhood Trauma Questionnaire. Valence was coded as negative (angry or fearful) stimulus = 0, positive (happy) stimulus = 1. Significant effects are highlighted in boldface (*p* < .05).
